# Supplementary material for: Modeling glioblastoma heterogeneity as a dynamic network of cell states
Source: Mol Syst Biol. 2021 Sep 16;17(9):e10105. doi: 10.15252/msb.202010105 (PMC8444284; doi:10.15252/msb.202010105)
Supplement: Supplementary file 6 — Source Data for Figure 5 [file MSB-17-e10105-s004.zip › Figure5A_sourcedata/GSEA_3071/hallmarks_stateVI.GseaPreranked.1623742557031/HALLMARK_OXIDATIVE_PHOSPHORYLATION.html]

Details for gene set HALLMARK\_OXIDATIVE\_PHOSPHORYLATION[GSEA]

|  || Dataset | state63071 |
| Phenotype | NoPhenotypeAvailable |
| Upregulated in class | na\_pos |
| GeneSet | HALLMARK\_OXIDATIVE\_PHOSPHORYLATION |
| Enrichment Score (ES) | 0.21292843 |
| Normalized Enrichment Score (NES) | 1.33926 |
| Nominal p-value | 0.054054055 |
| FDR q-value | 0.13820821 |
| FWER p-Value | 0.64 |
Table: GSEA Results Summary

  

Fig 1: Enrichment plot: HALLMARK\_OXIDATIVE\_PHOSPHORYLATION      
 Profile of the Running ES Score & Positions of GeneSet Members on the Rank Ordered List

  

| PROBE | GENE SYMBOL | GENE\_TITLE | RANK IN GENE LIST | RANK METRIC SCORE | RUNNING ES | CORE ENRICHMENT || 1 | LDHA |  |  | 197 | 0.604 | -0.0303 | Yes |
| 2 | MRPS15 |  |  | 202 | 0.602 | -0.0068 | Yes |
| 3 | TIMM9 |  |  | 224 | 0.586 | 0.0114 | Yes |
| 4 | MRPS30 |  |  | 240 | 0.571 | 0.0306 | Yes |
| 5 | MRPL15 |  |  | 307 | 0.537 | 0.0341 | Yes |
| 6 | TIMM17A |  |  | 370 | 0.502 | 0.0374 | Yes |
| 7 | GRPEL1 |  |  | 379 | 0.497 | 0.0555 | Yes |
| 8 | HCCS |  |  | 396 | 0.491 | 0.0711 | Yes |
| 9 | GLUD1 |  |  | 433 | 0.476 | 0.0806 | Yes |
| 10 | MDH2 |  |  | 434 | 0.476 | 0.1001 | Yes |
| 11 | ATP5MC1 |  |  | 494 | 0.457 | 0.1023 | Yes |
| 12 | ATP5PB |  |  | 518 | 0.450 | 0.1143 | Yes |
| 13 | COX5A |  |  | 528 | 0.449 | 0.1302 | Yes |
| 14 | CYCS |  |  | 612 | 0.422 | 0.1242 | Yes |
| 15 | SDHB |  |  | 629 | 0.416 | 0.1368 | Yes |
| 16 | MRPL35 |  |  | 659 | 0.410 | 0.1455 | Yes |
| 17 | ATP5F1B |  |  | 762 | 0.384 | 0.1327 | Yes |
| 18 | TOMM22 |  |  | 789 | 0.379 | 0.1410 | Yes |
| 19 | ATP6V1D |  |  | 822 | 0.372 | 0.1473 | Yes |
| 20 | SLC25A3 |  |  | 828 | 0.372 | 0.1611 | Yes |
| 21 | CPT1A |  |  | 832 | 0.371 | 0.1755 | Yes |
| 22 | OXA1L |  |  | 903 | 0.361 | 0.1707 | Yes |
| 23 | VDAC2 |  |  | 1025 | 0.341 | 0.1508 | Yes |
| 24 | CYC1 |  |  | 1042 | 0.338 | 0.1602 | Yes |
| 25 | NDUFA9 |  |  | 1067 | 0.335 | 0.1672 | Yes |
| 26 | IDH3B |  |  | 1079 | 0.333 | 0.1778 | Yes |
| 27 | HSPA9 |  |  | 1087 | 0.333 | 0.1895 | Yes |
| 28 | COX11 |  |  | 1113 | 0.328 | 0.1959 | Yes |
| 29 | MRPS12 |  |  | 1131 | 0.326 | 0.2045 | Yes |
| 30 | SLC25A5 |  |  | 1163 | 0.321 | 0.2090 | Yes |
| 31 | FH |  |  | 1237 | 0.311 | 0.2014 | Yes |
| 32 | VDAC1 |  |  | 1362 | 0.296 | 0.1788 | Yes |
| 33 | UQCRB |  |  | 1402 | 0.292 | 0.1799 | Yes |
| 34 | TIMM50 |  |  | 1405 | 0.291 | 0.1912 | Yes |
| 35 | FXN |  |  | 1425 | 0.288 | 0.1977 | Yes |
| 36 | ATP5MC3 |  |  | 1454 | 0.285 | 0.2016 | Yes |
| 37 | MTRR |  |  | 1460 | 0.284 | 0.2118 | Yes |
| 38 | ECHS1 |  |  | 1498 | 0.280 | 0.2129 | Yes |
| 39 | NDUFS8 |  |  | 1670 | 0.263 | 0.1759 | No |
| 40 | ATP5F1C |  |  | 1691 | 0.261 | 0.1810 | No |
| 41 | ACAT1 |  |  | 1709 | 0.260 | 0.1869 | No |
| 42 | PDHA1 |  |  | 1750 | 0.255 | 0.1861 | No |
| 43 | IDH3A |  |  | 1765 | 0.254 | 0.1926 | No |
| 44 | NDUFA7 |  |  | 1841 | -0.255 | 0.1821 | No |
| 45 | ATP6V0C |  |  | 2021 | -0.282 | 0.1436 | No |
| 46 | ALDH6A1 |  |  | 2091 | -0.294 | 0.1364 | No |
| 47 | ECI1 |  |  | 2195 | -0.313 | 0.1205 | No |
| 48 | NDUFA3 |  |  | 2216 | -0.317 | 0.1279 | No |
| 49 | BAX |  |  | 2397 | -0.353 | 0.0920 | No |
| 50 | IDH1 |  |  | 2526 | -0.384 | 0.0719 | No |
| 51 | NNT |  |  | 2583 | -0.396 | 0.0725 | No |
| 52 | MAOB |  |  | 2666 | -0.415 | 0.0666 | No |
| 53 | SLC25A4 |  |  | 2697 | -0.425 | 0.0756 | No |
| 54 | ATP6AP1 |  |  | 2779 | -0.449 | 0.0713 | No |
| 55 | ATP5F1E |  |  | 2811 | -0.461 | 0.0815 | No |
| 56 | COX17 |  |  | 2879 | -0.486 | 0.0827 | No |
| 57 | GPX4 |  |  | 2913 | -0.499 | 0.0940 | No |
| 58 | IDH2 |  |  | 2966 | -0.524 | 0.1009 | No |
| 59 | COX6C |  |  | 3054 | -0.565 | 0.0997 | No |
| 60 | ATP1B1 |  |  | 3245 | -0.711 | 0.0757 | No |
| 61 | PDP1 |  |  | 3362 | -0.826 | 0.0771 | No |
Table: GSEA details [plain text format]

  

Fig 2: HALLMARK\_OXIDATIVE\_PHOSPHORYLATION: Random ES distribution      
 Gene set null distribution of ES for **HALLMARK\_OXIDATIVE\_PHOSPHORYLATION**
